# Computational Image and Molecular Analysis Reveal Unique Prognostic Features of Immune Architecture in African Versus European American Women with Endometrial Cancer

**DOI:** 10.21203/rs.3.rs-3622429/v1

**Published:** 2023-12-29

**Authors:** Anant Madabhushi, Sepideh Azarianpour-Esfahani, Sirvan Khalighi, Arpit Aggarwal, Vidya Viswanathan, Pingfu Fu, Stefanie Avril

**Affiliations:** Emory University; Case Western Reserve University; Georgia Institute of Technology and Emory University; Georgia Institute of Technology and Emory University; Georgia Institute of Technology and Emory University; Case Western Reserve University; Case Western Reserve University

**Keywords:** Immune architecture, Tumor-infiltrating lymphocytes, Racial disparity, Digital pathology, Endometrial cancer

## Abstract

Endometrial cancer (EC) disproportionately affects African American (AA) women in terms of progression and death. In our study, we sought to employ computerized image and bioinformatic analysis to tease out morphologic and molecular differences in EC between AA and European-American (EA) populations. We identified the differences in immune cell spatial patterns between AA and EA populations with markers of tumor biology, including histologic and molecular subtypes. The models performed best when they were trained and validated using data from the same population. Unsupervised clustering revealed a distinct association between immune cell features and known molecular subtypes of endometrial cancer that varied between AA and EA populations. Our genomic analysis revealed two distinct and novel gene sets with mutations associated with improved prognosis in AA and EA patients. Our study findings suggest the need for population-specific risk prediction models for women with endometrial cancer.

## Introduction

Endometrial cancer (EC) is a prominent gynecologic malignancy in the United States. annually affecting around 66,200 women and representing the second most-prevalent cancer among the female population^1^. The majority of EC cases belong to type I tumors, primarily endometrioid, often diagnosed at an early stage facilitating curative interventions^2^. However, a subset of EC, specifically type II or non-endometrioid neoplasms, which are high-grade and often deeply invasive lesions, exhibits higher aggressiveness and an increased likelihood of relapse, posing challenges for treatment and displaying poor prognosis^2,3^.

Recent data highlight a concerning racial disparity in EC survival rates, particularly with African American (AA) women facing a significantly higher mortality risk compared to European American (EA) women^4^. The 5-year mortality rate stands at 39% for AA women and 20% for EA women^5^, drawing attention to multifaceted disparities rooted in systematic racism, structural biases, and cultural influences^6^. Studies have shown that delayed diagnoses among AA women contribute to this higher mortality, along with lifestyle choices and comorbid conditions^7–9^. Although AA women more often present with advanced stages of disease, higher grade tumors, and more aggressive type II tumors, their survival is significantly lower for all tumor types even after stratifying by age, stage, and grade^10–14^.

In studies where all participants had equal access to surgical care, adjuvant chemotherapy, and radiation therapy, AA women, even after adjusting for treatment and prognostic factors, demonstrated poorer survival rates compared to other racial groups^15^. Moreover, among AA women with EC and equal healthcare access, there was a higher prevalence of aggressive non-endometrioid histology types, such as serous carcinoma and carcinosarcoma in comparison to EA populations^16^. The AA group also exhibited significantly increased rates of advanced stage and high grade tumors when compared to EA patients^6,9^.

However, all the above mentioned clinical and social/demographic factors together fail to wholly explain the observed racial disparities in outcome, indicating the involvement of further biological, molecular, and/or immunological factors.

Biological differences, including genetic susceptibilities, seem to significantly contribute to the observed health disparities. Studies^17,18^ have linked the genetic predisposition of AA populations to more aggressive subtypes in breast and prostate cancers; in endometrial cancer, AA women are more frequently diagnosed with aggressive subtypes, such as serous and clear cell carcinomas, which are associated with a heightened risk of recurrence, advanced metastasis, and increased overall mortality^19^. Furthermore, molecular differences between populations can lead to variations in the characteristics of benign and malignant neoplasms^20^, which may be reflected in the morphological features seen in tissue pathology images and influencing the cancer outcomes^21^. For example, molecular designations, such as high copy number alterations (CNH), low copy number alterations (CNL), microsatellite instability (MSI), and polymerase-epsilon (POLE) mutations are distributed unevenly between AA and EA patients; these disparities can indeed impact disease progression and outcomes^22^.

Beyond molecular and genomic changes, differences in the tumor microenvironment play an important role in endometrial carcinogenesis^23^. More specifically, the immune cell architecture within EC is poorly understood to date and may be crucial for comprehending the impact of the tumor microenvironment on survival outcomes and disparities in gynecologic cancers. The interaction between tumor-infiltrating lymphocytes (TILs) and cancer cells is recognized as pivotal in the immune response within variably immune-rich tumors like non-small cell lung cancer or breast cancer^24,25^. Computational image analysis and machine learning (ML) approaches have emerged as valuable tools for quantitatively describing the immune milieu from (H&E) slides, providing information for survival outcomes and immune responses in gynecologic cancers^24^. Several methods^26,27^ have been established to evaluate the density of TILs. Notably, correlations have been observed between survival outcomes in gynecologic cancers with machine-based quantification of the number, density, and distribution of TILs. Additionally, stroma has been identified as a significant factor in predicting clinical outcomes and characterizing lymphovascular invasion in cancer metastasis^28–30^. Therefore, studying the immune milieu, particularly in the stromal region, could provide valuable insights into population differences and racial disparities in survival outcomes of gynecological cancers.

In this study, we employ computerized image and bioinformatic analysis to address three-tiered objectives. Firstly, employing, computational image analysis and machine learning techniques, we aim to discern molecular and quantitative structural variances within the endometrial cancer microenvironment in H&E tissue images, specifically focusing on distinctions between AA and EA women. Secondly, we aim to construct predictive models for the respective AA and EA populations, integrating morphologic features, molecular biomarkers, and self-reported race data to forecast progression-free survival (PFS). Finally, we also interrogate the association between stromal morphology descriptors and the underlying tumor biology and molecular subtypes, evaluating the presence of molecular and genomic distinctions between AA and EA populations.

## Results

### Differential tumor characteristics and outcomes in AA and EA populations

The Cancer Genome Atlas (TCGA) data was divided into a training set (T_0_) and an internal test set (T_1_) using a 2:1 random split. Additionally, the University Hospitals (UH) data was used as an external test set (T_2_). Test sets T_0_, T_1_, and T_2_ were also analyzed in population-based subsets, namely T_0EA_, T_0AA_, T_1EA_, T_1AA_, T_2EA_, and T_2AA_ (Methods).

T_0_, T_1_, and T_2_ were examined to investigate the impact of histology, grading, stage, and molecular designation (CNH, CNL, MSI, and POLE) within AA and EA populations. In T_0EA_, T_1EA_, and T_2EA_ women with EC were more likely to be diagnosed with the endometrioid subtype, classified as a type I tumor, and known to be associated with favorable outcomes^3^. On the other hand, T_0AA_, T_1AA_, and T_2AA_ had a higher proportion of poorer prognostic histology, such as mixed and serous subtypes, which are associated with increased mortality and recurrence rates^15^ (**Extended Data Fig. 1a** and **1b**). There is also a higher prevalence of high-grade and advanced-stage cancer cases among individuals of the AA cohort (**Extended Data Fig. 1c**-**1f**). CNH genomic subtypes characterized by high copy number alterations often coincide with the high-grade serous subtype of EC and are more prevalent in AA (**Extended Data Fig. 1g** and **1h**). The fraction bar plots, generated using the raw clinical data from **Extended Data Table 1**, illustrate the distributions of clinical variables including histology, grade, stage and molecular subtype among AA and EA patients.

### Evaluating population-specific versus population-agnostic prognostic models in AA and EA women

Population-specific models M_AA_ and M_EA_ were developed utilizing T_0AA_ and T_0EA_, respectively, while the population-agnostic model M_PA_ was developed using the entirety of T0. All three models utilized immune cell architecture descriptors from H&E-stained slides to predict PFS, assigned distinct risk scores to patients, and employed finely-tuned thresholds to categorize them into delineated risk groups. Ultimately, M_AA_, M_EA_, and M_PA_ were evaluated on T_1_, T_1AA_, T_1EA_, T_2_, T_2AA_ and T_2EA_.

**Figure 1:**
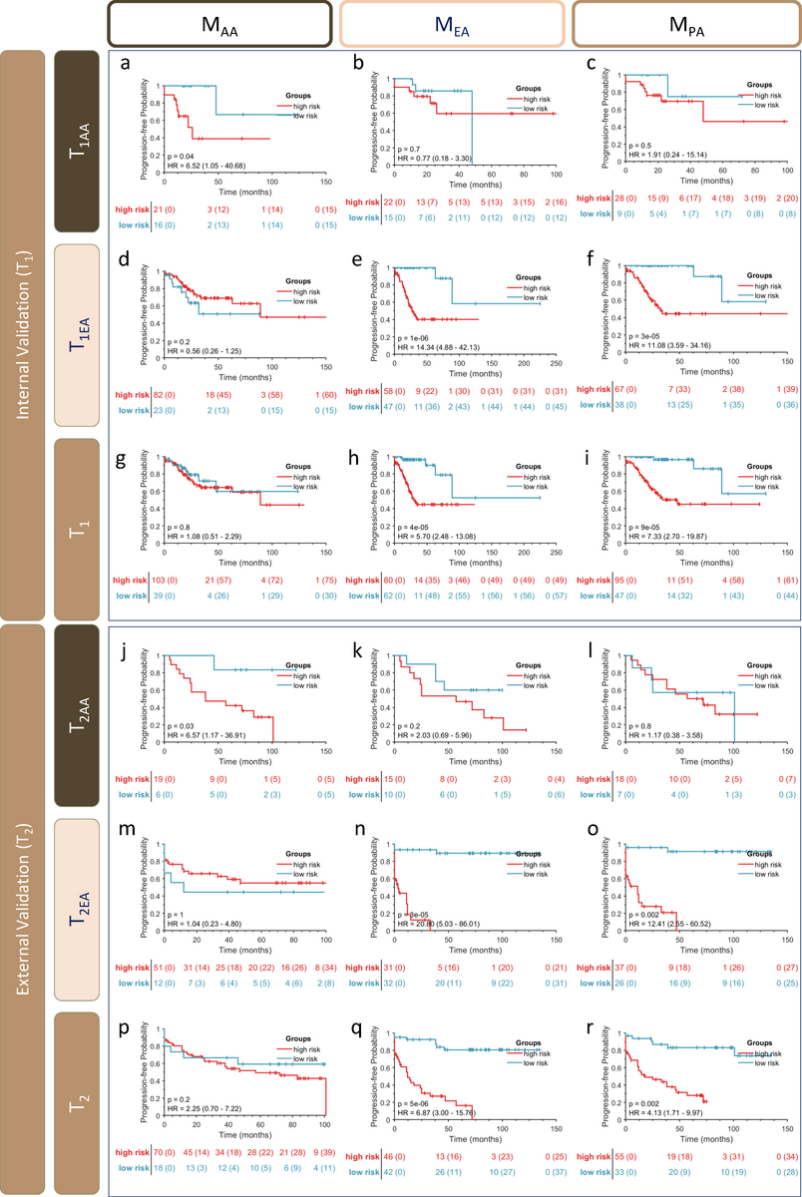
Kaplan-Meier curves for high- and low-risk groups. The Y-axis indicates the probability of PFS, and the X-axis represents time after treatment. Censored patients are shown as “+” marks. Stratification tables show the population at risk (censored) at each time point. Red and Blue curves denote High and Low-risk groups, respectively. Information about the p-value of the log-rank test, hazard ratios (HR) including their 95% confidence intervals (CI) of PFS time curves, for the TCGA cohorts (T_1_, **a-i**) and UH cohorts (T_2_, **j-r**).

Four prognostic features were identified in connection with M_AA_ relating to the interaction of stromal TIL clusters with surrounding stromal cell nuclei. Kaplan-Meier analysis of PFS in the delineated risk groups stratified using M_AA_ was described using the associated p-value, hazard ratio (HR), and 95% confidence interval (CI), all illustrated in Fig. 1. M_AA_ yielded a concordance (C) index of 0.86, 0.39 and 0.53 for T_1AA_, T_1EA_, and T_1_, respectively (Fig. 1a, 1d, and 1g). In T_2AA_, T_2EA_, and T_2_ M_AA_ demonstrated a C-index of 0.90, 0.50, and 0.67 respectively (Fig. 1j, 1m, and 1p).

The M_EA_ model comprised seven prognostic features, extracted from both epithelial and stromal regions. The M_EA_ exhibited a C-index of 0.70, 0.93, and 0.89 for T_1AA_, T_1EA_, and T_1_, respectively (Fig. 1b, 1e, and 1h). In T_2_, M_EA_ yielded a C-index of 0.50, 0.90, and 0.76 for T_2AA_, T_2EA_, and T_2_ (Fig. 1k, 1n, and 1q).

The M_PA_ model comprised six prognostic features, extracted from both epithelial and stromal regions. The M_PA_ exhibited a C-index of 0.48, 0.95, and 0.87 for T_1AA_, T_1EA_, and T_1_ respectively (Fig. 1c, 1f, and 1i). In T_2_, M_PA_ yielded a C-index of 0.76, 0.94, and 0.84 for T_2AA_, T_2EA_, and T_2_ (Fig. 1l, 1o, and 1r). M_PA_ effectively distinguished two risk strata within the EA population (T1_EA_, and T2_EA_), as well as in the population-blinded cohorts (T_1_ and T_2_). However, it was not prognostic in the AA subsets. Both the population-specific models, M_AA_ and M_EA_, were not prognostic when evaluated on populations for which they were not specifically designed.

Both univariate and multivariable Cox regression analyses demonstrated that population-specific risk scores of M_AA_ and M_EA_ were independently prognostic, even after accounting for clinicopathological variables (p < 0.05). This suggests that the immune architectural risk scores have a distinct influence on prognosis beyond the influence of patient characteristics or clinicopathological factors, **Extended Data Tables 5–6**. See **Supplementary Notes S.1**.

### Identifying population-specific immune morphologic image features

The prognostic features that influenced M_AA_, M_EA_, and M_PA_ were classified based on whether they were captured within the epithelial nests or the surrounding stromal compartment. Table 1 provides details of the contributing features of M_AA_, M_EA_, and M_PA_.

In M_AA_, all four features were associated with the morphology of the stromal compartment. Conversely, for M_EA_, only two out of seven features were derived from the stroma, while the remaining five were epithelium-related. Similarly, for M_PA_, two out of six features were derived from the stroma, with the remainder of the features coming from the epithelial region.

**Figure 2:**
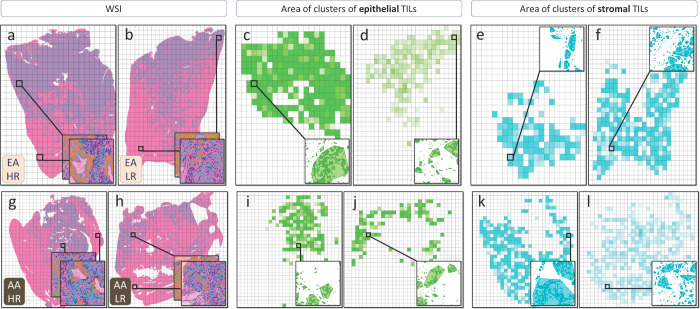
Spatial Distribution of TIL cluster area in the epithelium and stroma compartments for High-Risk and Low-Risk Pairs of AA and EA women. (**a-b**) The WSI heatmaps illustrate the distribution of the TIL cluster area, with intense colors representing higher values and faint colors indicating lower values of the feature per tile. Zoomed-in images emphasize the graphical representations and their convex hulls, used for calculating the area, with one epithelial and one stromal tile magnified arbitrarily for each patient. Comparing high-risk and low-risk EA cases (**c-d**), a notable increase in epithelial TIL cluster areas is observed in the high-risk case (**c**) in contrast to the low-risk case (**d**), while the stromal TIL areas remain relatively consistent (e-f). For the AA pair (**g-l**), a significant augmentation in stromal TIL cluster areas is evident in the high-risk case (**k**) compared to the low-risk case (**l**), while the size of epithelial TIL clusters remains relatively similar (**i-j**).

Upon reviewing the list of feature descriptions in Table 1, the feature “average area cluster of TILs,” contributed to both M_AA_ and M_EA_; however, they were captured in different tissue compartments. In the EA population, high-risk cases exhibited larger “epithelial” TIL clusters, while AA high-risk cases had larger “stromal” TIL clusters compared to low-risk cases. **Supplementary Notes S.2** and Fig. 2 provide detailed information on the compartmental region of origin for this feature, further elucidating its relationship with race-based characteristics.

### Investigating the molecular basis of differentially prognostic immune-related features between AA and EA populations

**Figure 3:**
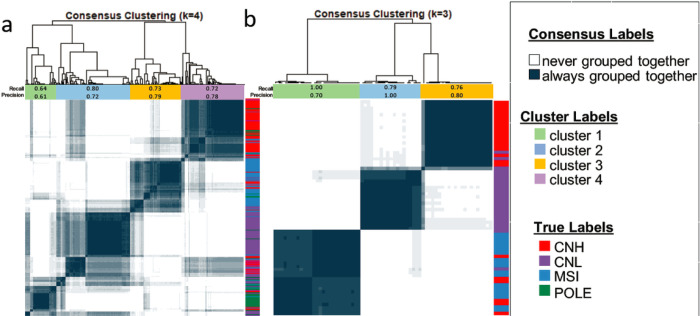
Clustergrams reveal distinct subgroups based on the preponderance of TIL profiles and genomic subtypes. left panel: TCGA cohort, right panel: UH cohort.

This experiment aims to explore the association between the graph-based TIL features and the genomic subtypes of EC. Carcinomas that are genomically characterized by CNH, CNL, MSI, and POLE mutations are recognized as distinct categories within EC and are associated with varying clinical outcomes^22^. Notably, there is a trend of improving outcomes from CNH to POLE, as demonstrated in **Extended Data Fig. 2**. An unsupervised clustering technique was employed to uncover potential associations between the EC genomic subtypes and the list of contributing morphological features to M_PA_. It was observed that the resulting clusters with distinct TIL profiles exhibited overlap with molecular/genomic tumor subtype. The recall (and precision) values for the T_0_ and T_1_ cohort were as follows: 0.64 (0.61) for the POLE group, 0.80 (0.72) for the CNL group, 0.73 (0.79) for the MSI group, and 0.72 (0.78) for the CNH group (Fig. 3a). Similarly, in T_2_, the recall (and precision) values were as follows: 1.00 (0.70) for MSI, 0.79 (1.00) for CNL, and 0.76 (0.80) for CNH (Fig. 3b). These results suggest a strong association between the graph-based TIL features and the genomic subtypes.

To further explore the presence of the connection between the immune-related features and the genomic subtypes within population-specific models, the unsupervised clustering algorithm was applied to the feature values that were relevant to the corresponding population model (**Extended Data Fig. 3**). The recall (and precision) values for the T_0AA_ and T_1AA_ cohort were as follows: 0.50 (0.83) for the POLE group, 0.75 (0.57) for the CNL group, 0.81 (0.90) for the CNH group, and 0.90 (0.76) for the MSI group (**Extended Data Fig. 3a**). The recall (and precision) values for the T_0EA_ and T_1EA_ cohort were as follows: 0.67 (0.74) for the POLE group, 0.77 (0.74) for the CNH group, 0.68 (0.82) for the MSI group, and 0.87 (0.76) for the CNL group (**Extended Data Fig. 3b**). The recall (and precision) values for the T_2AA_ cohort were as follows: 0.57 (0.80) for the MSI group, 1.00 (1.00) for the CNL group, and 0.92 (0.79) for the CNH group (**Extended Data Fig. 3c**). The recall (and precision) values for the T_2EA_ cohort were as follows: 0.83 (0.71) for the MSI group, 0.77 (0.83) for the CNL group, and 0.86 (0.90) for the CNH group (**Extended Data Fig. 3d**). These findings further suggest that there might be potential connections between TIL profiles and genotypic characteristics across diverse populations.

### Prognostic gene signatures, mutated genes, and metabolic pathways associated with the population-specific immune-related risk score within AA and EA populations.

To explore the association between the spatial architectural patterns of cells within the TME and the presence of mutated genes in EC patients, we systematically categorized a subset of patients of the AA and EA cohorts from TCGA dataset (Fig. 5). Specifically, we considered patients with available somatic mutation annotations (**Supplementary Notes S3**) and H&E WSI. These patients were divided into two distinct groups, namely those harboring gene mutations and those exhibiting wild-type gene status (**Supplementary Notes S3**).

**Figure 4:**
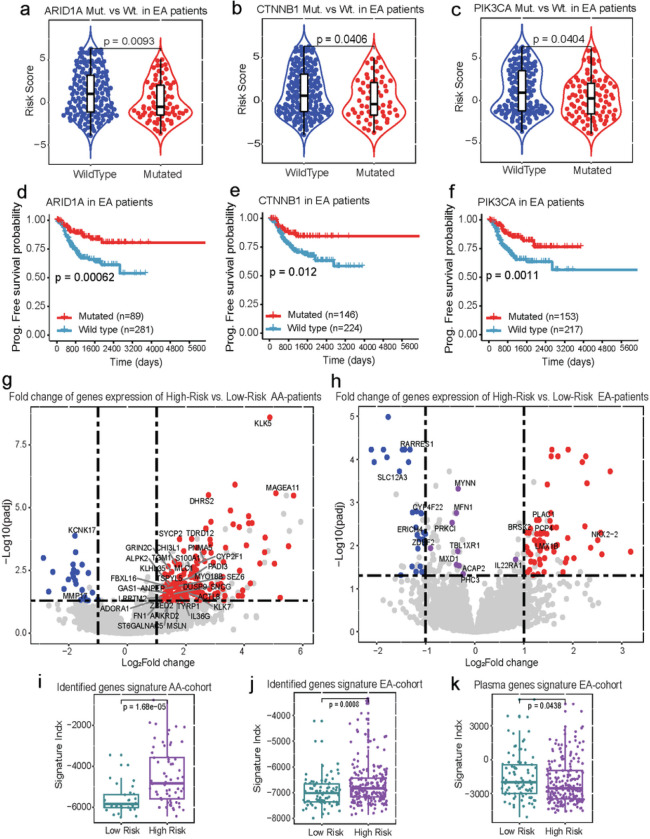
Genomics association and prognostic significance of spatial architectural patterns of immune and cancer cells in AA and EA cohorts. **a-c** Box-violin plot depicting significant difference of the predicted risk between EA patients harboring mutation in *ARID1A, CTNNB1*, and *PIK3CA* as compared to wild-type counterparts, respectively. **d-f** Shown are Kaplan–Meier estimates of PFS for patients harboring a mutation in *ARID1A* (**d**), *CTNNB1* (**e**), and *PIK3CA*(**f**), respectively. The statistical significance of differences in survival rates between mutated and wildtype categories was determined using the Log-rank test (P). **g-h** Volcano plot depicting significant differential expression between high-risk and low-risk patients across AA patients (**g**) and EA patients (**h**). X-axis denotes the difference (log2fold-change) of expression between high-risk and low-risk patients, whereas the Y-Axis denotes the statistical significance (−log10FDR) of the difference assessed using a Wilcoxon test. Points above the horizontal black dashed line denote genes inducing a significant shift (log10FDR<0.01) in their expression between high-risk and low-risk patients. Upregulated protein-coding genes with a fold change greater than one are highlighted with red dots, while downregulated genes are highlighted with blue dots. Additionally, protein-coding genes that are highly correlated with the predicted risk scores are labeled on the plot. **i-k** Box plots detailing the activity levels of the identified gene signatures in AA (**i**), the identified gene signatures in EA (**j**), and Plasma cells in EA (**k**) for high-risk compared to low-risk patients. The statistical significance of differences in the signature activities was estimated using a Wilcoxon signed rank test.

Our analysis revealed that in the EA cohort, non-synonymous somatic mutations in the *ARID1A, CTNNB1*, and *PIK3CA* genes were associated with a diminished risk of recurrence, as well as a favorable prognosis (Fig. 4a–c), while in the T_0AA_ and T_1AA_ cohort, no significant association was found between the non-synonymous somatic mutations of the driver genes and risk of recurrence or death. Furthermore, the Kaplan-Meier estimates of PFS illustrate concordant patterns of favorable survival for the samples harboring non-synonymous somatic mutations in these genes compared to their wild-type counterparts (Fig. 4d–f).

To unravel underlying biological mechanisms associated with morphological immune features, we started by identifying a subset of genes whose expressions exhibited significant correlations with estimated risk scores derived from M_AA_ and M_EA_ (Pearson correlation P<0.05). Subsequently, for each cohort of AA and EA patients, we conducted an analysis of differentially expressed genes to identify those that exhibited the most significant differences between high- and low-risk patient groups identified by M_AA_ and M_EA_ risk scores. The selection of the most important genes was based on a false discovery rate (FDR) threshold of <0.05 and a log2-fold change threshold of >1 (**Supplementary Notes S4**). Our investigation unveiled two distinct sets of genes for AA and EA, that showed significant correlations with risk and displayed differential expression patterns between the high-risk and low-risk patient groups (Fig. 4g–h, **Extended Data Table 2** and **3**).

We then employed single-sample gene set enrichment (ssGSEA) methodology to estimate the activity index (**Supplementary Notes S4**) of the distinct gene signatures specific to each population^31^. The results revealed that the identified AA-specific genes signature (**Extended Data Table 4**) was significantly up-regulated in high-risk AA patients compared to low-risk patients (Wilcoxon p=1.68e−05) (Fig. 4i). Similarly, the estimated activity index of the identified subset of EA-specific gene signatures (**Extended Data Table 4**) showed a significant upregulation in high-risk EA patients (Wilcoxon p=0.0008) (Fig. 4j). Upon testing some of the predefined gene’s signatures, the plasma cell gene signature (**Extended Data Table 4**) exhibited a significant downregulation in EA patients (Wilcoxon p= 0.0438) (Fig. 4k).

Furthermore, we conducted an in-depth investigation into the association between the top-identified genes from the AA and EA models and patients’ survival. Remarkably, among the identified genes, we discovered that certain genes, including *ADORA1, CHI3L1, CYP2F1, FN1, KLK7, S100A, ST6GALNAC5, SYCP2*, and *TDRD12*, exhibited significant prognostic implications for PFS and OS in only AA patients (**Extended Data Fig. 4a-i and 5a-i**). Conversely, these genes did not exhibit prognostic significance for either PFS or OS within the EA cohort (**Extended Data Fig. 6a-i and 7a-i**). On the other hand, *PRKCI* was found to be associated with PFS and OS exclusively in EA patients (**Extended Data Fig. 4j and 5j**) but not in AA patients (**Extended Data Fig. 6j and 7j**).

## Discussion

Even though race is a complex construct with socio-political implications, capturing cultural and demographic facets^32,33^, racial disparities in various gynecological cancers result in higher mortality rates among African American (AA) women compared to other populations^4,34^. AA women with EC face a twofold higher mortality rate over a 5-year span, revealing an uneven burden compared to EA women^34^.

While socio-economic factors may contribute to disparities in AA women with EC^8^, biological factors, including high-grade serous carcinoma subtypes, advanced diagnosis stages, and increased recurrence risk, are also influential. The most aggressive molecular subtype of endometrial cancer, CNH, is more prevalent in AA compared to EA women^21^. Recent studies have identified specific genetic variations, more prevalent in AA women, and linked them to poor prognosis of these patients^35^. Previous research in other types of cancer, including studies by Kim et al. in breast cancer^18^ and Bhargava et al. in prostate cancer^17^, have also confirmed the existence of such genomic and race-specific variations. These studies revealed that population-tailored models were needed to be able to predict more accurate outcomes in AA patients compared to EA patients.

In this study, we performed a comprehensive computational assessment of both morphological and molecular differences in EC. First, using computer vision and image analysis techniques, we aimed to discern and quantify variations in the structural arrangement of cells within the EC microenvironment between AA and EA women. Second, we identified distinct gene sets associated with population-specific risk scores, displaying significant differential expression patterns between high-risk and low-risk EC patient groups. Furthermore, we endeavored to construct population-specific prognostic models for these two racial groups, exploring the potential integration of pathomics biomarkers and racial data to predict PFS. Lastly, we set out to investigate whether morphological descriptors of the stromal tissue are associated with tumor biology and molecular subtypes, with a particular emphasis on elucidating molecular and genomic disparities between AA and EA populations.

Prognostic immune architecture descriptors integrated with tumor biology markers unveil morphological and molecular differences between AA and EA women in EC. Our genomic analysis identified *ARID1A*, *CTNNB1*, and *PIK3CA* gene mutations linked to favorable prognosis and lower recurrence risks in EA patients. Furthermore, mutations in these genes are more prevalent in endometrioid histologic subtypes and CNL genomic subtypes of endometrial cancers. These genes are key players in vital cellular processes, including chromatin remodeling, Wnt signaling, and PI3K-AKT-mTOR pathways, respectively, suggesting altered functionality^36–38^. The favorable prognosis associated with these mutations suggests potential functional alterations that confer a survival advantage to tumor cells, leading to a less aggressive disease course.

Furthermore, our study revealed distinct gene sets associated with population-specific risk scores, displaying significant differential expression patterns between high-risk and low-risk EC patient groups. Particularly, *ADORA1, CHI3L1, SYCP2, CYP2F1, FN1, KLK7, S100A, ST6GALNAC5*, and *TYRP1*, exhibited strong correlations with PFS and OS in AA patients but not in EA patients. Conversely, *PRKCI* emerged as significantly correlated with PFS and OS only in EA patients. Interestingly, *PRKCI’*s expression promotes an immune suppressive microenvironment that contributes to poor prognosis in human cancers, and elevated *PRKCI* expression is associated with immune-suppressive TME and poor prognosis in various cancers^39^. Although the roles of these genes in EC are not extensively explored, a literature review yielded insight. *ADORA1*^40,41^, *CHI3L1*^42^, *FN1*^43,44^, and *KLK7*^45^ were reviewed. Yet, the functions of other identified genes, linked to poorer survival and disease progression due to upregulation, remain enigmatic, necessitating further investigation.

These genes demonstrate strong predictive value in assessing both long-term outcomes, as seen in their impact on OS, and short-term endpoints like treatment efficacy and disease progression, as evidenced by their lower PFS p-values (indicating more precise stratification) (**Extended Data** Fig. 4–7).

The molecular alterations in the EC observed between populations lead to morphologic variations in tissue images as well^35^. It is noteworthy that many of these genomic differences originate in the tumor’s surrounding stroma^23^. Consequently, they can potentially impact the quantitative histomorphometry of the TME^16,18^. Our study revealed distinctive morphological variations in tumor epithelial nests and stroma between AA and EA women with EC. These differences encompass spatial arrangement and co-localization of TILs and tumor cells (Table 1).

Analyzing TIL-tumor cell interactions, our goal was to uncover distinct predictive patterns for PFS in each population. We assessed 125 AA and 392 EA women using AI-assisted tools for image analysis. Proximal cell graphs yielded computationally characterized immune architecture features from neoplasm H&E WSIs, predicting prognosis with population-specific (M_AA_, M_EA_) and population-agnostic (M_PA_) models. Our study showed that a consistent TIL architecture was prognostic for EC progression. Yet, significance varied across populations and races. Population-specific models outperformed in their prognostic ability, highlighting race-specific immune patterns. However, population agnostic model M_PA_ was prognostic only in the EA subset but not in AA, possibly due to higher EA cases. This underscores the importance of considering racial variations and sample size disparities when interpreting model performance. After correcting for batch effects and ensuring that the differences hold consistently across multiple sites, the same prognostic value was observed for population-specific models. Univariate and multivariate analyses revealed that this immune-related morphological risk was prognostic of PFS independent of clinical variables (**Extended Data Tables 5 and 6**).

Our finding of significant differences in the molecular and histological aspects of EC cases among AA and EA populations aligns with a recent study^46^. However, our research goes beyond merely highlighting variations in outcomes and aims to uncover the key triggers specific to each population. The density of TILs and immune cells in the stroma has been shown to be a quantitative characterization of the TME and correlated with an improved prognosis on patient outcomes in several types of cancer, including breast, colorectal, and lung cancer^24,25^. These studies have demonstrated that high tumor-stromal content, which is an indicator of the presence of cancer-associated fibroblasts, reflects the inflammation profile of the tumor and thus causes an adverse clinical outcome. Also, the presence of lymphovascular invasion can often be characterized by stroma^30^. Our observation implies an important role of morphology and architecture of TILs in the stromal compartment in prognosticating PFS in AA women with EC, while epithelial TIL features were more prognostic in EA women. Previous research, such as the study by Bhargava et al.^17^, has highlighted morphologic differences in stromal cell features between AA and EA men across different stages of prostate cancer as well. These differences have been linked to the higher incidence and mortality rates observed in AA men with prostate cancer. They utilized computational biology and population-specific information, including image descriptors from stromal regions, to enhance prognosis accuracy in AA men with prostate cancer through AI-assisted pathology-driven models. Similar observations were seen in our study, where we revealed population-specific morphologic differences in disease phenotypes, corroborating the importance of tailoring models. The analysis of contributing features in our study showed that the feature “average area cluster of TILs” represents a similar concept, but in different compartments (AA: stroma, EA: epithelium). This implies that the final signature may exhibit the same cell pattern, captured in different tissue compartments.

Our study has several limitations that should be acknowledged. First, we only focused on TILs and did not account for other immune cell types that play critical roles in remodeling the TME, such as tumor-associated macrophages, fibroblasts, and collagen^47,48^. Additionally, it is important to consider the distinct roles of different subtypes of TILs. Complementary data beyond H&E staining, such as immunohistochemistry, is necessary to fully understand immune modulation^49^. Moreover, while population-tailored prognostic models partially address concerns related to EC disparities, comprehensive solutions should involve addressing systemic issues, such as racism and unequal access to healthcare. Furthermore, validation of the genomic variations observed in larger and more diverse cohorts is crucial for the reliability and generalizability of our findings. In conclusion, this study provides a comprehensive exploration of immune architectural disparities in EC between AA and EA women, shedding light on potential molecular and biological factors contributing to these differences.

Our research findings hold relevance for advancing precision oncology approaches, with the ultimate goal of improving outcomes in EC patients and addressing the pressing issue of racial disparities in cancer care. Unfortunately, several extant risk prediction models have been shown to not be equitable across different populations. A notable example is Oncotype DX, a prognostic and predictive gene-expression assay that was recently shown to not be prognostic in black women^50^. This underscores the urgency of refining and diversifying precision medicine assays to ensure inclusivity across diverse patient populations. The integration of population-specific risk stratification and immune-related gene signatures opens new avenues for targeted therapies and enhanced patient management, ultimately leading to more effective and equitable care for individuals affected by endometrial cancer.

We acknowledge that race is a complex socio-political and legal construct, and the growing mixed-race population in the United States^7^ further highlights the intricacies of defining populations based on traditional racial categories. Nevertheless, our findings suggest that morphologic differences do exist among populations. While there are limitations to using race as a defining factor, it is increasingly apparent that moving forward, we should explore more tailored risk prediction models that consider a broader range of factors beyond traditional racial classifications.

In conclusion, our research represents a significant step towards achieving better precision medicine in gynecologic oncology. It serves as a compelling reminder of the imperative need to create more inclusive and effective predictive assays, reflecting the diversity of patient populations, and emphasizes the importance of continued efforts to bridge the gap in cancer care, ensuring equitable access and improved outcomes for all EC patients.

## Methods

### Dataset Description

A total of 517 patients were included in this study. Hematoxylin and eosin (H&E)-stained slide images from post-surgery EC patients were obtained from The Cancer Genome Atlas (TCGA) and University Hospitals Medical Center, Cleveland, Ohio (UH). Only patients with self-reported races of AA or white (EA) were included, while those with unknown or other races were excluded. Standard treatment with primary surgery was administered to all patients, followed by adjuvant radiation or chemotherapy for those considered intermediate or high risk of recurrence. The TCGA cohort comprised 429 patients, and with a total of 486 slides. Among these patients, 392 had a single WSI, while the remaining 37 patients with larger tumors had multiple slides, ranging from two to eight WSIs. In the UH cohort, each patient had two or three slides. The data from TCGA were scanned using Aperio ScanScope, while the UH cases were scanned using the Ventana medical scanner, resulting in 225 slides. Both cohorts were scanned at 0.2527 microns per pixel (MPP) resolution.

**Figure 5:**
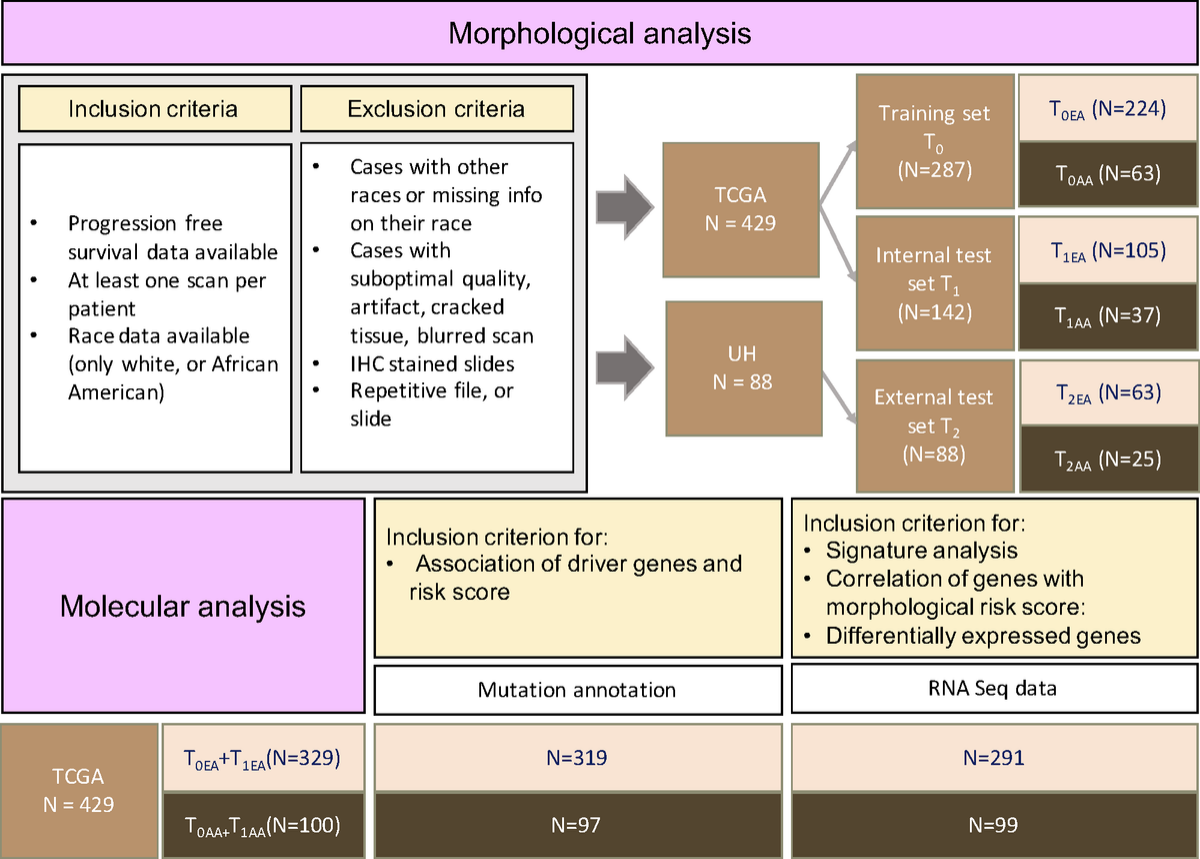
Data breakdown and training-validation split. Shown is the breakdown of patient data by race, with African American (AA) and European American (EA) cohorts. The TCGA cohort was divided into training (T_0_) and internal test (T_1_) sets for each racial group. The UH data served as the external test set (T_2_). Population-specific models were developed within each racial group, while a population-agnostic model was developed on the entire TCGA training data. The subset of TCGA patients with mutation annotations was utilized for analyzing the association of driver genes and the morphological risk score. The other subset of TCGA patients with available RNASeq data was incorporated in the signature analysis, identifying the correlation between genes and morphological risk scores, and identifying differentially expressed genes.

Population-specific models were developed utilizing the corresponding racial training groups and evaluated on respective racial validation groups with the ( - random splitting (Fig. 5)), while a population-agnostic model was developed using the entire racial groups within training data and evaluated on the whole validation data. Using immune cell architecture descriptors extracted from H&E-stained slides, the two population-specific prognostic models (M_AA_ and M_EA_) and a population-agnostic model (M_PA_) were trained to predict PFS. Each model identified a distinct set of features with differential prognostic values specific to their respective training cohorts.

WSIs were used to create non-overlapping tiles for feature analysis. To ensure the quality of the tiles, a quality assessment tool, HistoQC^51^, was employed. This tool helped identify unsuitable-quality tiles, such as those that were blurred, had cracked tissue portions, or contained artifacts from the scanning process. No WSI was excluded from the study due to HistoQC. Patients with multiple whole-slide scans usually had particularly large tumor masses, necessitating the storage of multiple files. In this study, any available scans for these patients were included, and tiles were extracted from each of those scans.

### Tissue Phenotyping

Tissue phenotyping entails the identification and separation of distinct tissue compartments, followed by the segmentation of individual cell types and their subsequent classification. This process allows for a comprehensive analysis of tissue composition. To automatically segment tissue into epithelial and stromal compartments, WSIs at 10x magnification were tiled (**Extended Data Fig. 8a and 8d**) and processed by a deep learning model that was trained and validated using annotated oral cavity tissue microarray images^52^. The deep learning model architecture incorporated a U-Net^53^, consisting of 28 layers and 14,788,929 parameters.

In this step of the algorithm, a grayscale image with the same dimensions as the input image tile is generated. Each pixel in the output tile is assigned a value indicating the probability of it belonging to the epithelium. This probabilistic mask is then converted into a binary mask using a threshold determined by qualitative input from our collaborating expert pathologist (**Supplementary Notes S.5**). The binary mask is post-processed by removing connected components with a total area smaller than 2,000 pixels (equivalent to 125 square microns) using morphological operations. For the parameters in this step, we also use input from the human expert. The resulting binary masks are filtered using the binary mask from HistoQC^51^ to ensure that no low-quality components interfere with the downstream framework. This step is illustrated in **Extended Data Fig. 8b** (whole slide view) and **Extended Data Fig. 8e** (zoomed tile view). Tiles within non-empty patches of epithelial nests or stroma were deemed regions of interest, demarcated in **Extended Data Fig. 8c**.

Nuclei segmentation was performed using the Hover-Net model, a state-of-the-art algorithm for nuclei segmentation and classification^54^. After segmentation, an SVM image-driven model^55^ was applied for TIL detection. Nuclei features, including shape, color, and texture, were used to classify each segmented nucleus as TIL or non-TIL. Following the classification, cell nuclei contours were reduced to their centroids. The coordinates of four different cell families, including stromal non-TILs, stromal TILs, epithelial non-TILs, and epithelial TILs, were utilized to extract spatial graph features (**Extended Data Fig. 8f**). The performance of the nuclei segmentation task was also qualitatively evaluated by an expert pathologist to ensure adequate identification of the nuclei regions. For more expansive details and qualitative considerations, see (**Supplementary Notes S.5**).

### Capturing quantitative immune profiles

The quantitative immune profile of TIL and non-TIL cells within epithelial nests and their surrounding stroma was characterized by cell cluster subgraphs, introduced in^15^. This method measures the spatial infiltration profile, cancer cell, and TIL interplay, and their relative density by the construction of cell subgraphs using a predetermined threshold distance, followed by the calculation of the relative abundance and spatial proximity of cells within each region (**Supplementary Notes S.6**). Utilizing the cell-type subgraphs, we generated a total of 350 quantitative features (**Extended Data Table 7)** for each tile within a region of interest (ROI). These features describe either a single cell type (**Extended Data Fig. 9a-b and 9d-e**) or the relationship between TILs and non-TIL cells (**Extended Data Fig. 9c and 9f**). We analyzed all the tiles within the ROI in WSI(s) captured for a patient. We then statistically analyzed the per-tile feature vector using six statistics (mean, median, minimum, maximum, range, and variance) on all stromal and epithelial tiles represented for a patient, yielding 4,200 features per patient.

### Model Construction

The TCGA AA and EA cohorts were randomly divided with the - random splitting into training and internal test sets, as described in Fig. 5. Population-specific models (M_AA_ and M_EA_) were developed using T_0AA_ and T_0EA_ data and evaluated on corresponding racial groups (T_1AA_ and T1EA), while a population-agnostic model was developed using the entire TCGA training data (T_0_) and evaluated on the entire TCGA validation data (T_1_). The UH data (T_2_), served as the external validation cohort in two different settings: 1) as a whole (T_2_) to evaluate the performance of the population-agnostic model, and 2) in separate racial groups (T2_AA_ and T2_EA_) to evaluate the performance of population-specific models.

### Genomic and Bioinformatics Analysis

A set of 14 numerical values was constructed for each patient by merging all unique spatial TIL features that contributed to M_AA_, M_EA_, and M_PA_. To identify distinct subgroups of patients based on these features, a robust unsupervised clustering method, namely the consensus clustering algorithm, was employed. By aggregating clustering results from multiple clustering runs and/or subsets of the data, the consensus clustering algorithm successfully identified multiple subgroups of patients with distinct TIL profiles. Patient stratification had a strong preponderance with known molecular and genomic designations. For TCGA (T_0_ + T_1_), the number of clusters is forced to four to match four different genomic profiles present within this cohort, and for UH (T_2_), the number of subsets was forced to three, to match three different genomic profiles available within this cohort. In this experiment, all the patients with missing molecular designations were excluded.

### Survival Analysis and Feature Selection

A Cox proportional hazard regression model was used to create population-specific prognostic models (M_AA_ and M_EA_) and a population-agnostic model (M_PA_) to predict PFS, trained on T_0AA_, T_0EA_, and T_0_. All three models were evaluated on T_1_ and T_2_ and their population subsets (T_1AA_, T_1EA_, T_2AA_, and T_2EA_).

To perform feature selection and train regression models of M_AA_ and M_EA_, we employed PFS time and all 4,200 patient-level immune architectural features of T_0AA_ and T_0EA_ datasets. For the population-agnostic model (M_PA_), the same steps were performed on the entire T_0_ dataset.

To ensure that the contributing factors were comparable, we standardized the features using the z-score method, based on their mean and standard deviation. This enabled us to facilitate a more straightforward comparison of the relative importance of each feature in the model.

The least shrinkage and selection operator (LASSO), in conjunction with the Cox proportional hazards regression model, was used to create PFS-prognostic models of M_AA_, M_EA_, and M_PA_^56^. The LASSO scheme identified only the most prognostic features for inclusion in the models. Features with small contributions were penalized by the sum of absolute values of their contribution factors, causing them to vanish to zero. This resulted in the vector of contributing factors being a sparse vector. By minimizing the partial likelihood of deviance in a 10-fold cross-validation fashion, the contributing features and their corresponding weights were obtained for M_AA_, M_EA_, and M_PA_. As a result, a risk score was created per each model by constructing a weighted linear combination of their prognostic features. Also, three finely-tuned thresholds (Th_AA_, Th_EA_, and Th_PA_) corresponding to each model were calculated in a way that maximizes hazard ratios between two risk strata in its specific training dataset. The dichotomized risk scores were employed to stratify the EC patients with high or low risk of disease progression.

M_AA_, M_EA_, and M_PA_ were evaluated to prognosticate the PFS outcome of the EC patients on the internal validation set, T_1_ (also T_1AA_ and T_1EA_) and external validation set, T_2_ (also T_2AA_ and T_2EA_) using the Kaplan-Meier method with the log-rank test to ensure that the models are adequately prognostic of PFS, and risk strata are statistically differential. The effect size of risk scores was estimated using hazard ratio (HR) and corresponding 95% confidence interval (CI) along with two-sided p-values (with < 0.05 being statistically significant), and concordance indices (C-index).

## Data Availability

All H&E WSI and genomic data from TCGA cohorts (T_0_ and T_1_) were derived from the TCGA Research Network: http://cancergenome.nih.gov/ and The Cancer Imaging Archive: https://www.cancerimagingarchive.net/. The H&E WSI data for the UH cohort (T_2_) is not currently permitted in public repositories because ethical and legal implications are still being discussed at the institutional level.
